# Technique of Suprapubic Prostatectomy

**Published:** 1919-03

**Authors:** G. Kolische

**Affiliations:** Attending Surgeon to the G. U. and Radio-Therapeutic Departments, Michael Reese Hospital, Chicago


### ORIGINAL ARTICLES.

### Technique of Suprapubic Prosetatectomy.

G. KOLISCHER

Attending Surgeon to the G. U. and Radio-Therapeutic Departments, Michael Reese Hospital, Chicago.

The technique of this operation has to answer two demands; reasonable safety of life, and the abolition of the inconvenience and the dangers of urinary obstruction. The primary mortality will be reduced to a negligible quantity by careful preoperative preparation of the patient, by keeping the hemorrhage during the operation in very narrow limits, and by checking it thoroughly and lastingly after the enucleation is completed, and finally by punctilious after-treatment. The functional results will be assured by avoiding any unnecessary traumatism and by avoiding the destruction of structures adjacent the tumefaction but not involved in the pathology. The preparation of the patient points at bringing him to the table in the best condition obtainable, which is accomplished by attending to the teeth and gums of the patient, by regulating the functions of the respiratory organs, of the heart, of the bowels, and of the kidneys. The indiscriminate use of cathartics has to be cautioned against, while the urine is acidified by the administration of mineral acids per mouth. The bladder is regularly and completely emptied by catheterization and following antiseptic flushing. As part of the preparation may also be considered the occasional necessary preliminary cystostomy.

This step may have to be employed, if one has to deal with prostatism plus infiltrating cystitis, or if the patient be suffering from pronounced Urosepsis, or if the kidneys on account of stagnation and backpressure are involved to such an extent that drainage of the bladder has to be established for some time, until the kidneys have sufficiently recovered to permit of the execution of the second step, the enucleation of the hypertrophied part of prostate.

The details of the prostatectomy are now arranged as follows: about twenty minutes previous to the beginning of the anesthesia, around either of the thighs as closely as possible to the inquinal fold, and around either of the arms as high as possible near the axillae an elastic band is placed just tightly enough so as not to compress the big arteries but to produce venous stasis only. In this way about one-third of the available blood supply is stored away in the four extremities. This leads to contraction of the superficial and intestinal arteries reducing considerably the bleeding during the operation. Incidentally, the quantity of the anesthetic necessary for insensibility is markedly diminished. The autotransfusion following the release of the constriction after the operation helps the patient to recover very quickly from the anesthetical stupor. Following the application of the tourniquets a Barnesbag is introduced into the rectum, taking care to spread it well underneath the prostate, and the bag is now distended by the injection of about one-hundred cc of water, after which its tube is clamped off. By this tamponing of the rectum the prostate and the trigonum is sufficiently elevated to make both structures easily accessible after once the bladder is opened.

Now the bladder is emptied through a catheter and refilled with 300 cc of a 2 per cent Protargol Solution.

The distension of the bladder facilities identifying if after the abdominal wall is split, while the flooding of the field of operation with the Protargol solution after opening the viscus helps to counteract any infection which the contents of the bladder may transmit to the wound. After removal of the catheter the penis is tied up with a strip of gauze in order to prevent any leakage and to insure the distension of the bladder during the steps necessary for its exposure. The patient is placed in Trendelenborg position and an incision is now made in the linea alba strating from the symhysis and extending upward for four to five inches. This incision just penetrates the skin exposing the subcutaneous fat. Out of the center of this wound a lump of fat of the size of a walnut is dissected out, which fat is later on to be used as a transplant into the bleeding cavity resulting from enucleation of the gland.

The fascia is now split with the knife and the recti muscles are separated bluntly.

In the wound appears the bulging bladder covered, as a rule, with the peritoneal reduplication. The latter is stripped off by gauze dissection until the anterior aspect of the bladder is well exposed. The bladder wall proper is recognized by its brownish color, by the muscular net work and by the large veins embedded in it. The viscus is now opened with a pointed knife, the operator cutting from above downward so as to prevent any slipping or plunging in the peritoneum. In case the serosa should have been accidentally opened during the stripping off of the peritoneum, the slit is closed with catgut sutures, before the bladder is opened.

The filling fluid emanating from the incision is now mopped up with large sponges, and the interior of the bladder is exposed by insertion of retractors, as such vaginal retractors being successfully employed. The proper insertion of the retractors deserves of some special attention. It is paramount to exert the traction in the occipito-caudal direction, and not as so often is done, in a frontal direction, because if the latter method is used a successful cleaning of the trigonum is almost impossible. But if one retractor is inserted in the upper angle of the wound, and the other is resting against he symphyseal angle, a free exposure of the bladder base is easily accomplished by slight traction. Whatever of the filling fluid remained in the bas fond is now removed by sponges attached to eight inch clamps. It may not be superfluous to mention that any sponging inside of the bladder ought to be done by dabbing and not by wiping, because the latter manipulation traumatizes the very vulnerable muccos and leads to unnecessary bleeding. Two landmarks are now recognized on the prostatic protrusion; one is the internal urethral orifice, and the other one a circular groove around the base of the prostatic tumor, indicating the course of the internal sphincter.

Both these structures ought to be handled with utmost care, in order to prevent the development of a traumatic stricture or of incontinence. Anywhere between these two landmarks the mucosa is now incised with a pointed knife to the extent of about two inches. This incision is deepened until the central prostatic tissue presents itself marked by its yellowish color in contradistinction to the dark red of the mucos. A finger tip palpating this slit will feel whether there are any more moveable layers over the hypertrophied part calling for a deepening of this cut or, whether the depth of the incision shows proper penetration through the surgical capsule. Boring with the fingernail through the mucosa and capsule or forcing a finer tip into the urethra and in this way to break through the capsule cannot be called proper procedure, failing to come up to the demand of modern surgery, that is to operate as much as possible under the guidance of the eye, and not to bruise tissues. After the proper cleavage is established a Kocher poker is inserted and the separation continued, until it is easy to insert the tip of the index finger into the separating gap. The retractors are now removed and by always working from behind forward, the hypertrophied part of the gland is now progressively detached from the capsule until the tumor hangs only on the urethra.

The insertion of two fingers into the rectum in order to facilitate the enucleation is objectionable, for the reason that in spite of changing of the glove infection from the rectum is liable to be carried into the wound. Such cases even with fatal ending are on record. If any counterpressure should be desired, it will be furnished by inserting a staff into the urethra from within the bladder.

By rolling the freed mass toward and over the symphysis the urethra is exposed and pulled taut and severed closely to the tumor with scissors or bitten through by finger pressure. A thorough palpation of the cavity follows this in order to discover and to remove any hypertrophied nodules, that may have been left. The retractors are now reinserted and the former bed of the prostrate is exposed. The upper angle of the trigonal wound is seized with an eight inch clamp carrying a sharp bit and the cavity is temporarily packed with a gauze strip, to check the hemorrhage until the operator gets ready to insert the transplant of fat, gleaned from the skin incision. The implanation of the fat is done in the following way: a small curved needle is armed with a long thread of catgut and the needle and thread are firstly carried through the right edge of the trigonal wound, then the needle is brought outside of the abdomen and made to perforate the lump of fat that is still kept outside, then the needle and sutures are carried again into the bladder, perforating now from within the other lip of the trigonal wound and brought again to the surface. By pulling on the ends of this suture the fat slips into the cavity, out of which now the packing is removed the fat is pressed down hard into the cavity and the suture is tied. In case there still should be some bleeding at the circumference of the transplant, a few additional sutures are inserted in the edges of the trigonal wound so as to press the plug of fat into intimate contact with the whole surface of the cavity. In case one has to deal with a prostate that was incarcerated underneath the symphysis, in consequence of which the distal corner of the trigonal wound is hard to reach, or if the plug having been cut too small and therefor oozing should persist in spite of the transplantation of the fat, definite hemostasis is obtained by packing with selvedged strips on top of the fatty transplant. The free end of the gauze strip is led out of the bladder alongside the rubber tube inserted for syphonage. This tamponade of the prostatic cavity is preferable to the insertion of a rubber bag or to the packing with gauze only, for this reason: bag or packing has to be removed after a certain time, and it is nothing unusual that this is followed again by a hemorrhage, highly dangerous and hard to deal with at this stage of the game. But the fat very soon becomes intimately attached to the cavity, and peripheral parts of the transplant become organized, while the bulk of the plug is evacuated through the drainage tube in form of oil drops and of detritus. Even if packing additional to the transplantation of fat had to be employed, the removal of this tamponade can be manipulated with safety, because at the time of the withdrawing of the gauze strip the fat is clinging securely to the raw surface, while the gauze does not stick to the fat so that a tearing off of the transplant and renewal of hemorrhage is not to be feared at the time of the removal of the gauze packing. After the hemorrhage is securely attended to a rubber tube of a diameter of about half an inch is inserted into the bladder and fastened to the upper angle of the wound with a purse string suture in such a way as to prevent the inner end of the tube from touching the base of the bladder in order to avoid unnecessary irritation. Then a very fine rubber drain is placed into the retrovesical space and the patient is brought back to the ordinary recumbent position. The rest of the bladder wound is closed by mattress sutures, the operator being careful to invert the mucosa and not let it get caught between the edge of the incision. Heavy tension sutures are now inserted through the skin and fascia, then the rectal fascia reunited by a running catgut suture, the skin wound is closed with silk or with clips and the tension sutures tied over a roll of gauze. The compression of the extremities and of the penis are now released, and the bag is emptied and removed out of the rectum. After the patient is placed in bed a glass connector bent at a right angle is inserted into the free end of the rubber tube. To the distal end of the glass connector, a rubber hose is attached, the free end of which dips into some antiseptic fluid contained in a large glass bottle, that is placed lower than the body of the patient. By injecting some fluid into this tube and submerging its free end a permanent syphonage of the bladder is established and maintained.

